# Application of Statistical Design for the Production of Cellulase by *Trichoderma reesei* Using Mango Peel

**DOI:** 10.1155/2012/157643

**Published:** 2012-12-06

**Authors:** P. Saravanan, R. Muthuvelayudham, T. Viruthagiri

**Affiliations:** Department of Chemical Engineering, Annamalai University, Annamalainagar 608002, Tamilnadu, India

## Abstract

Optimization of the culture medium for cellulase production using *Trichoderma reesei* was carried out. The optimization of cellulase production using mango peel as substrate was performed with statistical methodology based on experimental designs. The screening of nine nutrients for their influence on cellulase production is achieved using Plackett-Burman design. Avicel, soybean cake flour, KH_2_PO_4_, and CoCl_2_ *·*6H_2_O were selected based on their positive influence on cellulase production. The composition of the selected components was optimized using Response Surface Methodology (RSM). The optimum conditions are as follows: Avicel: 25.30 g/L, Soybean cake flour: 23.53 g/L, KH_2_PO_4_: 4.90 g/L, and CoCl_2_ *·*6H_2_O: 0.95 g/L. These conditions are validated experimentally which revealed an enhanced Cellulase activity of 7.8 IU/mL.

## 1. Introduction

The food and agricultural industries produce large volumes of wastes annually worldwide, causing serious disposal problems. This is more in countries where the economy is largely based on agriculture and farming practice is very intensive. Currently, these agrowastes are either allowed to decay naturally on the fields or are burnt. However, these wastes are rich in sugars due to their organic nature. They are easily assimilated by microorganisms and hence serve as source of potential substrates in the production of industrially relevant compounds through microbial conversion. In addition, the reutilization of biological wastes is of great interest since, due to legislation and environmental reasons, the industry is increasingly being forced to find an alternative use for its residual matter [1]. One of the agrowastes currently causing pollution problems is the peels of the mango (*Mangifera indica* L.) fruit. Mango is one of the most important fruits marketed in the world with a global production exceeding 26 million tons in 2004 [2]. It is cultivated or grown naturally in over 90 countries worldwide (mainly tropical and subtropical regions) and is known to be the second largest produced tropical fruit crop in the world [3]. The edible tissue makes up 33–85% of the fresh fruit, while the peel and the kernel amount to 7–24% and 9–40%, respectively [4].

In fact, mango peel as a byproduct of mango processing industry could be a rich source of bioactive compounds and enzymes such as protease, peroxidase, polyphenol oxidase, carotenoids, and vitamins C and E [5]. While the utilization of mango kernels as a source of fat, natural antioxidants, starch, flour, and feed has extensively been investigated [6, 7], studies on peels are scarce. Their use in biogas production [8, 9] or making of dietary fiber with a high antioxidant activity [10] has been described in the past. However, mango peels are not currently being utilized commercially in any way, though a large quantity is generated as waste (20–25% of total fruit weight) during mango processing thus, contributing to pollution [11].

Most studies on the exploitation of mango peels have been dealing with their use as a source of pectin, which is considered a high-quality dietary fiber, [12, 13]. Recently, a screening study of 14 mango cultivars had demonstrated the content and degree of esterification of mango peel pectins to range from 12% to 21% and 56% to 66%, respectively. Furthermore, mango peels have been shown to be a rich source of flavonol O-xanthone C-glycosides, gallotannins, and benzophenone derivatives [14]. However, reports on the use of mango peels for the production of industrially relevant metabolites such as lactic acid through fermentation processes are rare. Thus, cultivation of microorganisms on these wastes may be a value-added process capable of converting these materials, which are otherwise considered to be wastes, into valuable products through processes with technoeconomic feasibility.

With the increasing demand for alternative liquid fuels worldwide, cellulase is being used as the primary enzyme for enzymatic hydrolysis of lignocellulosic biomass in bioethanol production process. It is known that the production economics of bioethanol is largely dependent on the cost of cellulase. However, high cost of the enzyme presents a significant barrier to the commercialization of bioethanol. Therefore, finding an economic way to produce cellulase has drawn great attention around the world. The cost of enzymes is one of the main factors determining the economics of a biocatalytic process and it can be reduced by finding optimum conditions for their production. In order to minimize the enzymes production cost, considerable progress has been made in strain development, optimization of culture condition, mode of, and modelling the fermentation process [15].

Application of agroindustrial wastes in bioprocesses provides an alternative way to replace the refined and costly raw materials. In addition, the bulk use of such materials helps to solve many environmental hazards. However, the application of microorganisms for the production of cellulase using cost-effective raw materials is rare. Hence, research efforts are focused on looking for new and effective nutritional sources and new progressive fermentation techniques enabling the achievement of both high substrate conversion and high production [16].

In the present study, the screening and optimization of medium composition for cellulase production by *Trichoderma reesei *using Plackett-Burman technique in Response Surface Methodology (RSM) are carried out. The Plackett-Burman screening design is applied for identifying the significant variables that enhance cellulase production. The central composite design [CCD] was further applied to determine the optimum level of each significant variable.

## 2. Materials and Methods 

### 2.1. Raw Material

Mango peel of Alphonsa (king of mango) variety was collected by manually peeling off fresh undamaged ripe fruits purchased from a local fruit market in Salem, India. The underlying pulp on the peels was removed by gently scraping with the blunt edge of a clean knife and the peels were washed with distilled water to remove adhering dust.

### 2.2. Microorganisms and Maintenance

 The microorganism *Trichoderma reesei *NCIM 1186 is procured from National Chemical Laboratories, Pune, India. The strain was well preserved and cultured on potato dextrose agar (PDA) slants at 30°C for 5–7 days. They are then stored at 4°C during which there was formation of spores.

### 2.3. Inoculum Preparation

For inoculum preparation, 2.0 mL of a spore suspension (containing 10^8^ conidia/mL) of *T. reesei* was inoculated into 50 mL of the seed medium in a 250 mL Erlenmeyer flask. The content was cultured at a temperature of 30°C, pH of 5.5, and agitation speed of 180 rpm for three days.

### 2.4. Pretreatment

 The pretreatment process decreases the crystallinity of mango peel while removing lignin and other inhibitors there by enabling its enzymatic hydrolysis. 100 g of the washed ground mango peel was treated separately with 2000 mL of 2% NaOH solution and autoclaved at 121°C for 30 minutes. Then it was filtered, washed with distilled water, and excess alkali present was neutralized with phosphoric acid. Again it was filtered and the residue material was dried at 65°C in a hot air oven to constant weight. To the cellulosic material obtained, same volume of distilled water was added and heated at 121°C for 30 minutes. The suspension was filtered and the solid material was dried at 65°C in hot air oven [17]. The dried mango peel powder was used as a carbon source.

### 2.5. Fermentation Conditions

 Fermentation was carried out in 250 mL cotton plugged Erlenmeyer flasks with 10 g of pretreated mango peel at pH 7. This is supplemented with different nutrient concentration for tests according to the selected factorial design and sterilized at 120°C for 20 minutes. After cooling the flasks at room temperature, the flasks were inoculated with 1 mL of grown culture broth. The flasks were maintained at 30°C under agitation at 200 rpm for 48 hours. During the preliminary screening process, the experiments were carried out for 9 days and it was found that the maximum production was obtained at 6th day. Hence further experiments were carried out for 6 days.

### 2.6. Enzyme Assay

Cellulase activity (measured as filter paper hydrolysing activity, using a 1 × 6 cm strip of Whatman no. 1 filter paper) and cellobiase activity were assayed according to the method recommended by Ghose (1987) and expressed as international units (IU). One international unit of cellulase activity is the amount of enzyme that forms 1 *μ*mol glucose (reducing sugars as glucose) per minute during the hydrolysis reaction. Reducing sugar was determined by the dinitro salicylic acid (DNS) method [18].

### 2.7. Optimization of Cellulase Production

Plackett-Burman experimental design assumes that there are no interactions between the different variables in the range under consideration. A linear approach is considered to be sufficient for screening. Plackett-Burman experimental design is a fractional factorial design and the main effects of such a design may be simply calculated as the difference between the average of measurements made at the high level (+1) of the factor and the average of measurements at the low level (−1).

To determine the variables that significantly affect cellulase activity, Plackett-Burman design is used. Nine variables (Table 1) are screened in 20 experimental runs (Table 2) and insignificant ones are eliminated in order to obtain a smaller, manageable set of factors. The low level (−1) and high level (+1) of each factor are listed in (Table 1). The statistical software package Design-Expert software (version 7.1.5, Stat-Ease, Inc., Minneapolis, USA) is used for analysing the experimental data. Once the critical factors are identified through the screening, the central composite design is used to obtain a quadratic model.

### 2.8. Central Composite Design

The central composite design is used to study the effects of variables on their responses and subsequently in the optimization studies. This method is suitable for fitting a quadratic surface and it helps to optimize the effective parameters with minimum number of experiments as well as to analyse the interaction between the parameters. In order to determine the existence of a relationship between the factors and response variables, the collected data were analysed in a statistical manner, using regression. A regression design is normally employed to model a response as a mathematical function (either known or empirical) of a few continuous factors and good model parameter estimates are desired.

The coded values of the process parameters are determined by

(1) $$\begin{array}{c} x_{i}=\frac{X_{i}-X_{o}}{\Delta x}, \end{array}$$

where *X*_*i*_ is the coded value of the *i*_th_ variable, *X*_0_ is the uncoded value of the *i*_th_ test variable at center point and Δ*x* is the step change. The regression analysis is performed to estimate the response function as a second-order polynomial

(2) $$\begin{array}{c} Y=\beta_{0}+\sum_{i=1}^{k}\beta_{i}X_{i}+\sum_{i=1}^{k}\beta_{ii}X_{i}^{2}+\sum_{i=1,i<j}^{k-1}\sum_{\;j=2}^{k}\beta_{ij}X_{i}X_{j}, \end{array}$$

where *Y* is the predicted response, *β*_0_ constant, and *β*_*i*_, *β*_*j*_, and  *β*_*ij*_ are coefficients estimated from regression. They represent the linear, quadratic, and cross products of *X*_*i*_ and *X*_*j*_ on response.

### 2.9. Model Fitting and Statistical Analysis

The regression and graphical analysis with statistical significance are carried out using Design-Expert software (version 7.1.5, Stat-Ease, Inc., Minneapolis, USA). The minimum and maximum ranges of variables investigated are listed in (Table 3). In order to visualize the relationship between the experimental variables and responses, the response surface and contour plots are generated from the models. The optimum values of the process variables are obtained from the regression equation.

The adequacy of the models is further justified through analysis of variance (ANOVA) in Table 5. Lack-of-fit is a special diagnostic test for adequacy of a model and compares the pure error, based on the replicate measurements to the other lack of fit, based on the model performance. *F* value, calculated ratio between the lack-of-fit mean square, and the pure error mean square, these statistic parameters, are used to determine whether the lack-of-fit is significant or not, at a significance level.

## 3. Results and Discussions

**Figure 1:**
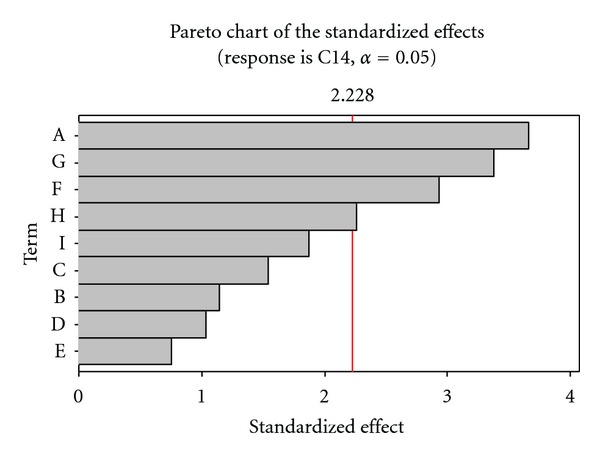
Pareto chart showing the effect of media components on cellulase activity (A-Avicel, F-Soybean cake flour, G-KH_2_PO_4_, and H-CoCl_2_·6H_2_O).

Plackett-Burman experiments (Table 2) showed a wide variation in cellulase production. This variation reflected the importance of optimization to attain higher productivity. From the Pareto chart (Figure 1) the variables, namely, Avicel, soybean cake flour, KH_2_PO_4_, and CoCl_2_·6H_2_O were selected for further optimization to attain a maximum response.

The level of factors Avicel, soybean cake flour, KH_2_PO_4_, and CoCl_2_·6H_2_O and the effect of their interactions on cellulase production were determined by central composite design of RSM. Thirty experiments were preferred at different combinations of the factors shown in (Table 4) and the central point was repeated five times (8, 10, 17, 20, 21, and 26). The predicted and observed responses along with design matrix are presented in (Table 4) the results were analysed by ANOVA. The second-order regression equation provided the levels of cellulase activity as a function of Avicel, soybean cake flour, KH_2_PO_4_, and CoCl_2_·6H_2_O, which can be presented in terms of coded factors as in the following equation:

(3) Y=7.80+0.36A+0.48B+0.53C+0.58D−0.28AB−0.063AC⁡−0.013AD+0.35BC+0.075BD+0.29CD−0.65A2−0.59B2−0.54C2−0.65D2,

where *Y* is the cellulase activity (IU/mL), *A*, *B*, *C*, and  *D* are avicel, soybean cake flour, KH_2_PO_4_, and CoCl_2_·6H_2_O, respectively. ANOVA for the response surface is shown in Table 4. The model *F* value of 14.74 implies the model is significant. There is only a 0.01% chance that a “Model *F* value” this large could occur due to noise. Values of “prob > *F*” less than 0.05 indicate model terms are significant. Values greater than 0.1 indicates model terms are not significant. In the present work, linear terms of *A*, *B*, *C*, *D*, and all the square effects of *A*, *B*, *C*, *D*, and the combination of *B*∗*C* and *C*∗*D* were significant for cellulase activity. The coefficient of determination (*R* ^2^) for cellulase activity was calculated as 0.93, which is very close to 1 and can explain up to 93.00% variability of the response. The predicted *R* ^2^ value of 0.70 was in reasonable agreement with the adjusted *R* ^2^ value of 0.86. An adequate precision value greater than 4 is desirable. The adequate precision value of 11.05 indicates an adequate signal and suggests that the model can be to navigate the design space.

**Figure 2:**
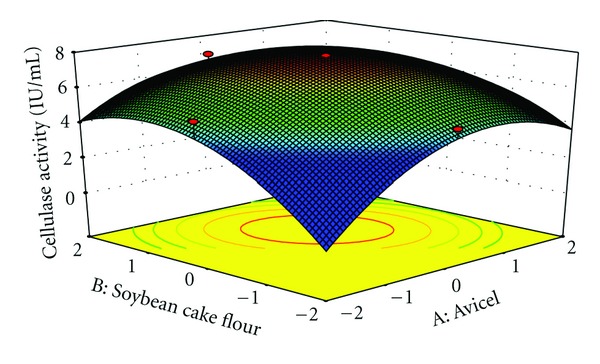
3D Plot showing the effect of Avicel and soybean cake flour on cellulase activity.

**Figure 3:**
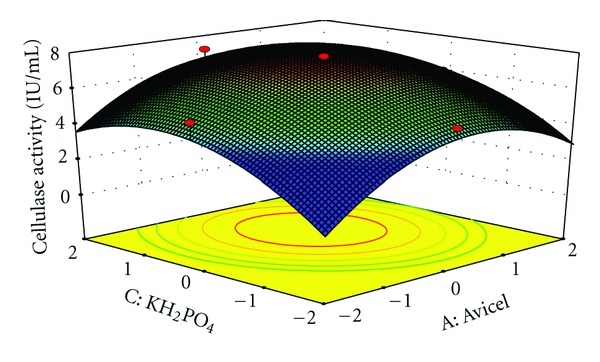
3D plot showing the effect of Avicel and KH_2_PO_4 _on cellulase activity.

**Figure 4:**
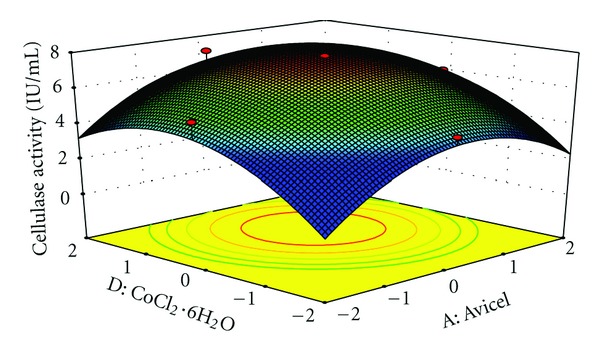
3D plot showing the effect of Avicel and COCl_2_·6H_2_O on cellulase activity.

**Figure 5:**
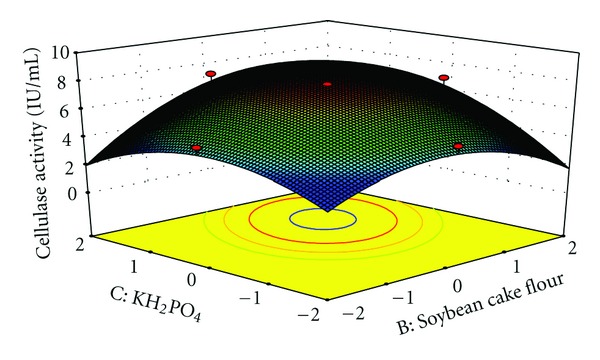
3D plot showing the effect of Soybean cake flour and KH_2_PO_4_ on cellulase activity.

**Figure 6:**
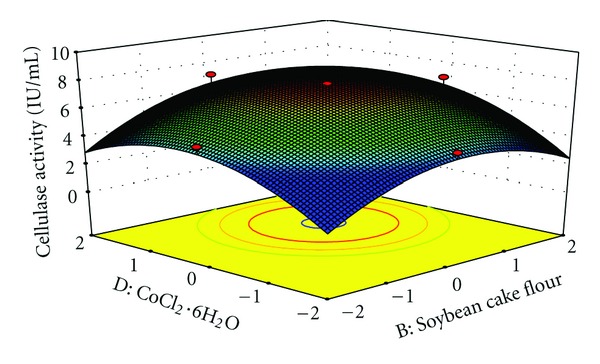
3D plot showing the effect of Soybean cake flour and CoCl_2_·6H_2_O on cellulase activity.

**Figure 7:**
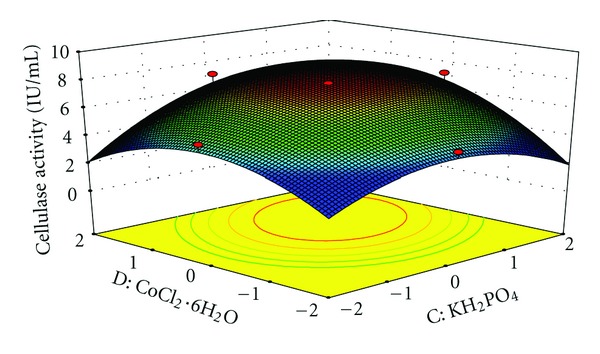
3D plot showing the effect of KH_2_PO_4_ and CoCl_2_·6H_2_O on cellulase activity.

The interaction effects of variables on cellulase production were studied by plotting 3D surface curves against any two independent variables, while keeping another variable at its central (0) level. The 3D curves of the calculated response (cellulase production) and contour plots from the interactions between the variables are shown in Figures 2, 3, 4, 5, 6, and 7.  Figure 2 shows the dependency of cellulase activity on avicel and soybean cake flour. The cellulase activity increased with increase in avicel to about 25.30 g/L and thereafter cellulase activity decreased with further increase in avicel. The same trend was observed in Figure 3. Increase in soybean cake flour resulted increase in cellulase activity up to 23.53 g/L which is evident from Figures 2 and 5. Figures 3 and 5 show the dependence of cellulase activity on KH_2_PO_4_. The effect of KH_2_PO_4 _on cellulase observed was similar to other variables. The maximum cellulase activity was observed at 4.90 g/L of KH_2_PO_4_. Figures 6 and 7 shows the dependency of cellulase activity on CoCl_2_·6H_2_O. The maximum cellulase activity was observed at 0.95 g/L.

### 3.1. Validation of the Experimental Model

Validation of the experimental model was tested by carrying out the batch experiment under optimal operation conditions: Avicel: 25.30 g/L, Soybean cake flour: 23.53 g/L, KH_2_PO_4_: 4.90 g/L, and CoCl_2_·6H_2_O: 0.95 g/L established by the regression model. Four repeated experiments were performed and the results are compared. The cellulase activity (7.8 IU/mL) obtained from experiments was very close to the actual response (7.84 IU/mL) predicted by the regression model, which proved the validity of the model.

## 4. Conclusions

In this work, Plackett-Burman design was used to determine the relative importance of medium components for cellulase production. Among the variables, avicel, soybean cake flour, KH_2_PO_4_, and CoCl_2_·6H_2_O were found to be more significant variables. From further optimization studies the optimized values of the variables for cellulase activity were found as Avicel: 25.30 g/L, soybean cake flour: 23.53 g/L, KH_2_PO_4_: 4.90 g/L, and CoCl_2_.6H_2_O: 0.95 g/L. This study showed the mango peel is a good source for the production of cellulase. Using the optimized conditions, the production reaches 7.8 IU/mL. 

## Figures and Tables

**Table 1:** Nutrients screening using Plackett-Burman design.

S. no.	Nutrients code	Nutrient	Minimum value g/L	Maximum value g/L
1	A	Avicel	15	35
2	B	Cornsteep flour	2	8
3	C	MnSO_4_·H_2_O	0.6	1.2
4	D	FeSO_4_·7H_2_O	0.7	1.3
5	E	Beef extract	20	40
6	F	Soybean cake flour	10	30
7	G	KH_2_PO_4_	2	6
8	H	CoCl_2_·6H_2_O	0.5	1
9	I	Yeast extract	5	15

**Table 2:** Plackett-Burman experimental design for nine variables.

Run order	A	B	C	D	E	F	G	H	I	Cellulase activity IU/mL
1	1	−1	−1	1	1	−1	1	1	−1	5.9
2	1	−1	1	−1	1	1	1	1	−1	7.2
3	1	1	1	−1	−1	1	1	−1	1	7.3
4	−1	−1	−1	−1	1	−1	1	−1	1	3.9
5	1	1	−1	−1	1	1	−1	1	1	6.1
6	1	1	1	1	−1	−1	1	1	−1	7.0
7	−1	−1	1	−1	1	−1	1	1	1	5.3
8	1	−1	−1	−1	−1	1	−1	1	−1	4.7
9	1	1	−1	−1	−1	−1	1	−1	1	6.0
10	−1	1	−1	1	1	1	1	−1	−1	5.5
11	−1	−1	1	1	−1	1	1	−1	−1	4.7
12	1	1	−1	1	1	−1	−1	−1	−1	4.0
13	−1	−1	−1	1	−1	1	−1	1	1	5.5
14	−1	1	1	−1	1	1	−1	−1	−1	3.8
15	−1	1	−1	1	−1	1	1	1	1	7.2
16	−1	1	1	−1	−1	−1	−1	1	−1	5.4
17	−1	−1	−1	−1	−1	−1	−1	−1	−1	2.4
18	−1	1	1	1	1	−1	−1	1	1	3.7
19	1	−1	1	1	−1	−1	−1	−1	1	5.1
20	1	−1	1	1	1	1	−1	−1	1	7.2

**Table 3:** Ranges of variables used in RSM.

S. no.	Variables	Code	−2	−1	0	+1	+2
1	Avicel	A	15	20	25	30	35
2	Soybean cake flour	B	10	15	20	25	30
3	KH_2_PO_4_	C	2	3	4	5	6
4	CoCl_2_·6H_2_O	D	0.4	0.6	0.8	1.0	1.2

**Table 4:** Central Composite Design (CCD) in coded levels with cellulase yield as response.

Run order	A	F	G	H	Experimental cellulase activity IU/mL	Predicted cellulase activity IU/mL
1	−1	1	−1	1	5.0	5.22
2	0	0	0	2	7.1	6.50
3	−1	−1	−1	1	3.8	4.27
4	1	−1	−1	−1	4.9	5.22
5	0	0	0	−2	4.6	4.16
6	0	−2	0	0	5.0	4.48
7	1	1	−1	1	5.1	5.49
8	0	0	0	0	7.8	7.80
9	1	−1	1	−1	4.7	4.89
10	0	0	0	0	7.8	7.80
11	0	2	0	0	6.9	6.38
12	1	1	1	1	7.5	7.70
13	1	−1	−1	1	5.6	5.64
14	0	0	2	0	7.2	6.70
15	−1	1	1	1	7.4	7.69
16	−1	1	1	−1	5.4	5.77
17	0	0	0	0	7.8	7.80
18	−1	−1	1	−1	3.5	3.72
19	−2	0	0	0	5.4	4.46
20	0	0	0	0	7.8	7.80
21	0	0	0	0	7.8	7.80
22	1	−1	1	1	6.4	6.45
23	−1	−1	−1	−1	3.6	3.80
24	2	0	0	0	6.0	5.90
25	1	1	−1	−1	4.9	4.77
26	0	0	0	0	7.8	7.80
27	0	0	−2	0	5.1	4.56
28	1	1	1	−1	5.7	5.84
29	−1	−1	1	1	4.8	5.34
30	−1	1	−1	−1	3.9	4.45

**Table 5:** Analyses of variance (ANOVA) for response surface quadratic model for the production of cellulose.

Source	Sum of square	df	Mean square value	*F* value	*P* value
Model	56.10	14	4.01	14.74	<0.0001
A-Avicel	3.08	1	3.08	11.33	0.0042
B-Soybean cake flour	5.41	1	5.41	19.92	0.0005
C-KH_2_PO_4_	6.83	1	6.83	25.11	0.0002
D-COCl_2_·6H_2_O	8.17	1	8.17	30.04	<0.0001
AB	1.21	1	1.21	4.45	0.0521
AC	0.063	1	0.063	0.23	0.06385
AD	2.500*E* − 003	1	2.500*E* − 003	9.195*E* − 003	0.09249
BC	1.96	1	1.96	7.21	0.0170
BD	0.090	1	0.090	0.33	0.5736
CD	1.32	1	1.32	4.86	0.0434
A^2^	11.74	1	11.74	43.17	<0.0001
B^2^	9.60	1	9.60	35.32	<0.0001
C^2^	8.05	1	8.05	29.60	<0.0001
D^2^	10.43	1	10.43	38.36	<0.0001
Residual	4.08	15	0.27		
Lack of fit	4.07	10	0.41		
Pure error	0.000	5	0.000		
Cor total	60.17	29			
